# Metagenomic Analysis of Anaerobic Microbial Communities Degrading Short-Chain Fatty Acids as Sole Carbon Sources

**DOI:** 10.3390/microorganisms11020420

**Published:** 2023-02-07

**Authors:** Daniela Becker, Denny Popp, Fabian Bonk, Sabine Kleinsteuber, Hauke Harms, Florian Centler

**Affiliations:** 1 UFZ—Helmholtz Centre for Environmental Research, Department of Environmental Microbiology, Permoserstr 15, 04318 Leipzig, Germany; 2 IAV GmbH, Kauffahrtei 23-25, 09120 Chemnitz, Germany; 3 Institute of Human Genetics, University of Leipzig Medical Center, Philipp-Rosenthal-Str. 55, 04103 Leipzig, Germany; 4 VERBIO Vereinigte Bioenergie AG, Thura Mark 18, 06780 Zörbig, Germany; 5 School of Life Sciences, University of Siegen, 57076 Siegen, Germany

**Keywords:** anaerobic digestion, syntrophic acetate oxidation, syntrophic propionate oxidation, syntrophic butyrate oxidation, methanogenic pathways, metagenome-assembled genomes, hybrid assembly

## Abstract

Analyzing microbial communities using metagenomes is a powerful approach to understand compositional structures and functional connections in anaerobic digestion (AD) microbiomes. Whereas short-read sequencing approaches based on the Illumina platform result in highly fragmented metagenomes, long-read sequencing leads to more contiguous assemblies. To evaluate the performance of a hybrid approach of these two sequencing approaches we compared the metagenome-assembled genomes (MAGs) resulting from five AD microbiome samples. The samples were taken from reactors fed with short-chain fatty acids at different feeding regimes (continuous and discontinuous) and organic loading rates (OLR). *Methanothrix* showed a high relative abundance at all feeding regimes but was strongly reduced in abundance at higher OLR, when *Methanosarcina* took over. The bacterial community composition differed strongly between reactors of different feeding regimes and OLRs. However, the functional potential was similar regardless of feeding regime and OLR. The hybrid sequencing approach using Nanopore long-reads and Illumina MiSeq reads improved assembly statistics, including an increase of the N50 value (on average from 32 to 1740 kbp) and an increased length of the longest contig (on average from 94 to 1898 kbp). The hybrid approach also led to a higher share of high-quality MAGs and generated five potentially circular genomes while none were generated using MiSeq-based contigs only. Finally, 27 hybrid MAGs were reconstructed of which 18 represent potentially new species—15 of them bacterial species. During pathway analysis, selected MAGs revealed similar gene patterns of butyrate degradation and might represent new butyrate-degrading bacteria. The demonstrated advantages of adding long reads to metagenomic analyses make the hybrid approach the preferable option when dealing with complex microbiomes.

## 1. Introduction

Biogas production by anaerobic digestion (AD) provides a sustainable option to transform organic waste into a renewable energy carrier. Under anoxic conditions, organic matter is converted to short-chain fatty acids (SCFAs) as intermediates, and finally to methane and carbon dioxide, catalyzed by different microbes in four steps: hydrolysis, acidogenesis, acetogenesis, and methanogenesis. The first three steps are facilitated by bacteria, whereas the last step is performed by archaea. There are multiple types of biogas reactors. The continuous stirred tank reactor (CSTR) is the most common type for agricultural biogas plants in Germany. The organic loading rate (OLR) is a key parameter in continuous AD processes. At high OLRs, large substrate amounts are fed to an AD reactor, and consequently high methane productivity can be expected. However, high OLRs can also lead to process instabilities and even process failure [1]. The stability of the AD process depends on the interaction of microbes of different functional groups [2]. An important prerequisite to control the process stability is to predict compositional and functional changes of the microbial community as a function of operational parameters. Community composition and complexity as well as operational parameters influence community dynamics [2,3]. For instance, the feeding regime can affect the process stability as well as the microbial community function [1,4,5,6]. The continuous feeding mode is commonly applied in full-scale digesters to obtain stable biogas production [7]. However, several studies showed positive effects of discontinuous feeding (e.g., one feeding per day) compared to continuous feeding [1,8,9,10]. Bonk et al. observed that discontinuous feeding leads to higher relative abundances of *Methanosarcina* [1]. This methanogen is more adapted to higher acetic acid concentrations and more resistant to low pH values compared to the common acetoclastic methanogen *Methanothrix* [5]. A higher share of *Methanosarcina* increased the resilience against organic overloading [1].

Some products of the acidogenesis and acetogenesis steps, such as acetate, formate, H_2_, and CO_2_ as well as methyl compounds, can be utilized by methanogenic archaea directly. In contrast, propionate and butyrate are acidogenesis products that first need to be converted into acetate, CO_2_, and H_2_ or formate [11]. In the whole AD process, syntrophic butyrate and propionate oxidation during acetogenesis is a bottleneck because of the low abundances of butyrate- and propionate-oxidizing bacteria and their sensitivity to environmental changes [2,12,13]. Disturbances of the AD process can lead to the accumulation of SCFA and consequently process instability [12,14,15,16]. Thus, the cooperation between bacteria and methanogenic archaea is essential for stable process performance. Typical propionate- and butyrate-oxidizing bacteria are *Syntrophobacter*, *Pelotomaculum, Smithella*, and *Syntrophomonas* [12,17]. In AD processes, hydrogenotrophic methanogens depend on hydrogen-producing bacteria, and inversely, syntrophic bacteria can only grow when the hydrogen partial pressure is kept low by hydrogenotrophs. For instance, fatty acid-degrading bacteria that use the β-oxidation pathway, such as *Syntrophomonadaceae*, grow in syntrophy with hydrogen-consuming microbes [6,18]. Besides acetoclastic methanogenesis catalyzed by *Methanothrix* or *Methanosarcina*, syntrophic acetate oxidation (SAO) is an alternative acetate sink in AD. Under mesophilic conditions, *Thermacetogenium phaeum*, *Pseudothermotoga lettingae*, *Tepidanaerobacter acetatoxidans*, *Clostridium ultunense*, and *Syntrophaceticus schinkii* [19] are key players of SAO whereby acetate is converted into H_2_ and CO_2_ [20,21]. In SAO, both the methyl and carboxyl groups of acetate are oxidized to CO_2_, and H_2_ is formed, but the reaction is unfavorable in the absence of hydrogenotrophic methanogens. Therefore, SAO is also an example of the interdependencies between bacteria and archaea in AD [22].

Two metabolic pathways for anaerobic propionate degradation have been described: the methylmalonyl-CoA pathway and the C6-dismutation pathway. Examples of syntrophic propionate-oxidizing bacteria (SPOB) that use the methylmalonyl-CoA pathway are *Syntrophobacter* and *Pelotomaculum* [23]. These SPOB degrade propionate directly to acetate and CO_2_. Propionate is first carboxylated to methylmalonyl-CoA, which is further converted via succinate, fumarate, and malate to oxaloacetate, followed by two decarboxylation steps to pyruvate and eventually to acetyl-CoA [24,25]. In contrast, *Smithella* uses the C6-dismutation pathway converting propionate to acetate and *n*-butyrate as an intermediate [26]. Butyrate is then further degraded to acetate via the β-oxidation pathway [27].

For the last AD step, methanogenesis, three important pathways have been described: the acetoclastic, the hydrogenotrophic, and the methylotrophic pathway. In the acetoclastic pathway, acetate is activated to acetyl-CoA and then cleaved into CO_2_ and a methyl group, which is further reduced to CH_4_ [28,29]. *Methanothrix* and *Methanosarcina* are the only two genera performing acetoclastic methanogenesis. Most of the other known methanogens utilize the hydrogenotrophic pathway, which accounts for the remaining portion of methane production resulting from the reduction of carbon dioxide with hydrogen/formate [30,31].

In this study, Illumina-based shotgun metagenome sequencing was used to characterize and compare compositional and overall functional profiles of reactor microbiomes being subjected to different feeding regimes and OLRs. By restricting the substrate to SCFA, we aimed to select microbiomes that are less diverse than common AD microbiomes and facilitate the functional analysis of acetogenesis and methanogenesis as the last two AD steps. To analyze the genetic profile of individual members of the microbial community (particularly the bacterial community), Illumina-based short-read and Oxford Nanopore-based long-read assemblies were compared with respect to the recovery of high-quality metagenome-assembled genomes (MAGs). Read length is a limitation of the Illumina platform but the high quality is an advantage. Oxford Nanopore reads are long, but the quality is much lower with an error rate of up to 15%, compared to the Illumina error rate of 0.473% (median) [32,33,34,35]. By applying a hybrid assembly approach, the disadvantages of both techniques can be compensated [36,37,38], hence we expected that the completeness of the MAGs increases compared to Illumina-based MAGs. Additionally, the identification and analysis of MAGs can help predict important functional groups [39,40,41].

## 2. Materials and Methods

### 2.1. Samples

The biogas reactor experimental design was described by Bonk et al. [1]. In brief, five reactor experiments were conducted (R-1 to R-5) using CSTRs with a working volume of 8 L (R-1 to R-3) or 6 L (R-4 and R-5) (total volume 15 L), which were operated under mesophilic conditions (37 °C), and with a hydraulic retention time of 8 days. Acetate, propionate and butyrate were fed as sole carbon sources in a synthetic medium in a ratio of 0.45:0.1:0.45, based on chemical oxygen demand (COD). This ratio reflects the product spectrum of acidogenic fermentation, in which typically more acetate and butyrate is formed than propionate (see e.g., [42]). Feeding was varied across two parameters: OLR (high and low) and feeding regime (continuous and discontinuous). Reactors R-1 and R-4 were continuously fed, while reactors R-2, R-3, and R-5 received a single feeding pulse per day (Table 1). All reactors were inoculated from a laboratory scale reactor that was fed with the same substrates. The original inoculum was derived from a lab-scale reactor digesting distillers grains.

All reactors had reached a stable production phase when sampling occurred (see Supplementary File S1 for details on observed process performance of all reactors). The following parameters were recorded: pH value, methane production rate, total SCFA concentration, and microbial biomass. The experimental setup, process parameters, and measured data in the stable production phase are summarized in Table 1.

For metagenome analysis using the Illumina MiSeq platform, 1.5 mL reactor effluents were centrifuged for 2 min at −7 °C and 15,000× *g*, immediately upon sampling. The supernatant was removed. The sample pellets for subsequent DNA extraction were stored at −20 °C. The DNA extraction followed the instruction (buffer SL2, no enhancer solution) of the NucleoSpin^®^ Soil Kit (MACHEREY–NAGEL GmbH & Co. KG, Germany). DNA purity was assessed with a NanoDrop One spectrophotometer (Thermo Fisher Scientific, Waltham, MA, USA), and for DNA quantification, the Qubit ™ dsDNA HS Assay Kit (Thermo Fisher Scientific, Waltham, MA, USA) was used.

### 2.2. Metagenome Sequencing

Libraries for whole-genome shotgun sequencing were prepared using the Nextera^®^ XT DNA Library Preparation Kit (Illumina, San Diego, CA, USA) according to the manufacturer’s protocol. Based on the quantification results by Qubit™ dsDNA HS Assay Kit (Thermo Fisher Scientific, Waltham, USA), the DNA samples were diluted to 0.2 ng/µL.

For indexing, the indexes i7 and i5 were used. Samples were purified using 30 µL AMPureXP beads (AGENCOURT^®^ AMPURE^®^ XP Kit) according to the manufacturer’s instructions. The purified library was eluted in 50 µL resuspension buffer. Finally, the library was checked by the Agilent 2100 Bioanalyzer (Agilent Technologies, Santa Clara, CA, USA) applying the Agilent High Sensitivity DNA Kit and by Qubit™ dsDNA HS Assay Kit (Thermo Fisher Scientific, Waltham, MA, USA).

The library pool was generated using the concentration (based on Qubit™ quantification) and the average size of DNA fragments (in bp) per sample (based on the Agilent 2100 Bioanalyzer quantification). An individual volume per library was pipetted to obtain a pool library of 50 nM, which was finally controlled using the Agilent High Sensitivity DNA Kit and Qubit dsDNA HS Assay Kit. Libraries including 5% PhiX DNA were sequenced on the Illumina MiSeq platform. For the reactors R-1 to R-3, the MiSeq Reagent Kit v2 (Illumina) with 500 cycles (2 × 250 bp reads) was used, and for the samples from reactors R-4 and R-5, the MiSeq Reagent Kit v3 (Illumina) with 600 cycles (2 × 300 bp reads) was used.

Extracted DNA was prepared for sequencing on the MinIon Mk1B platform (Oxford Nanopore Technologies, Oxford, UK) using the SQK-LSK108 Ligation Sequencing Kit (Oxford Nanopore Technologies, Oxford, UK) according to the manufacturer’s protocol. Libraries were loaded on rec V R9.4 flow cells. The MinIon device was operated with MinKNOW software v1.10.16.

Statistics for both sequencing approaches are provided in Supplementary File S2, B.1.

### 2.3. Bioinformatic Analysis

#### 2.3.1. Basecalling, Quality Filtering, and Read Trimming

For technical reasons, DNA from sample R-1 was sequenced in two technical replicates on the Illumina platform. Raw reads of both replicates from reactor R-1 were merged to increase the coverage. Adapter sequences of all reads were trimmed automatically during MiSeq sequencing.

First, the Illumina raw reads were quality-filtered by Trimmomatic v0.36 [43] to a minimum read length of 35 bp and minimum average quality of 20. Quality control before and after filtering was performed with FastQC [44] and compared using MultiQC [45]. Between 5 and 25 million high quality reads per sample were generated.

Basecalling of Nanopore raw reads was performed with Albacore v2.3.1 using default settings. Adapters were removed with Porechop v0.2.3 (https://github.com/rrwick/Porechop) using an identity threshold of 90% and splitting reads when adapters were found with 90% identity in the middle of a read. Quality control was performed with FastQC and MultiQC as well.

#### 2.3.2. Analysis of Community Composition and Functional Potential

Community composition was analyzed using a pipeline based on information of expected species of the AD process, as previously described by Becker et al. [46]. Expected species were chosen based on a literature search in the context of AD, gene prediction results using Metaxa v2.1.3 [47,48], and amplicon sequencing results from Bonk et al. [1], and combined in a reference database. The FASTA files including genomes of these expected species were included in this reference database [46]. According to the database, the reads were classified into expected species reads (aligned against the database) and unexpected species reads (unaligned). Bowtie2 v2.3.3.1 [49] was applied to align the Illumina reads against the database of expected species for the classification. Expected reads were directly annotated using DIAMOND v0.9.8 [50] and MEGAN6 v6.12.0 [51] based on the NCBI-NR database (accessed on 11 July 2018), whereas unexpected species reads were additionally subjected to de novo assembly and binning steps.

The assembly of unexpected reads was performed with IDBA v1.1.1 using the IDBA-UD command [52,53] and a k-mer range between 51 and 301, and binned with MaxBin v2.2.4 [54,55] using the marker gene set 40 specific for bacterial and archaeal communities. After binning, unexpected reads of each bin were reassembled using IDBA v.1.1.1 using the IDBA-UD command and a k-mer range between 51 and 301. Assembly and binning statistics were assessed with QUAST v4.5 [56] and CheckM [57]. Reassembled unexpected species reads were annotated with DIAMOND v0.9.8 [50] and MEGAN6 v6.12.0 [51] using the NCBI-NR database (accessed on 11 July 2018). Finally, the annotation results of expected and unexpected species were combined as described by Becker et al. [46].

The EggNOG database [58] option of DIAMOND/MEGAN5 was used to annotate the functional potential of expected reads and assembled unexpected reads by using the protein accession mapping file from October 2016. Finally, the read counts of annotations were combined to cover the whole functional potential (see Supplementary File S2, B.2.2).

To generate a functional catalog of all reads per sample, interleaved paired and unpaired reads were annotated together, without differentiating between expected and unexpected species reads as before. The databases used for the annotation with DIAMOND and MEGAN5 were the protein accession mapping file of EggNOG [58] from October 2016 and the GI to NCBI taxonomy mapping files for the NCBI-NR protein database from June 2018 (both available under http://ab.inf.uni-tuebingen.de/data/software/megan6/download/welcome.html, last accessed: 16 July 2018). Additionally, the taxonomy (genus level) and the functional potential (high-level nodes of EggNOG and Clusters of Orthologous Groups of proteins (COG) IDs) were combined to assess the functional potential per genus (R scripts available at https://git.ufz.de/UMBSysBio/mpp).

Based on the study by Sikora et al. [31], selected enzymes (genes) responsible for the relevant pathways of syntrophic butyrate, propionate, and acetate oxidation as well as methanogenesis, based on the Kyoto Encyclopedia of Genes and Genomes (KEGG) database, were collected (see Supplementary File S1, Tables S1 and S2). This list of genes was used to compile the functional catalog per pathway of interest (Supplementary File S2, B.5).

#### 2.3.3. Reconstruction and Annotation of Metagenome-Assembled Genomes

For reconstructing MAGs, the Illumina reads per sample of the continuously fed reactors (R-1 and R-4, without selection of expected/unexpected species reads) were assembled with SPAdes genome assembler v3.11.1 [59] using the metaSPAdes mode applying k-mers between 51 and 201, and binned with MaxBin v2.2.7 [54,55] using default settings (marker gene set 40). Assembly and binning statistics were generated with QUAST and CheckM, respectively. High quality MAGs (>90% completeness and <5% contamination) and medium quality MAGs (50–89.99% completeness and <10% contamination) [60] were refined with refineM v0.1.1 [61]. Thereby, potential contaminations were removed based on genomic properties, taxonomic assignments of contigs, and incongruent taxonomic assignment of 16S rRNA genes.

Next to the Illumina-based MAGs, Nanopore reads were included in the reconstruction of genomes. They were corrected and assembled with Canu v1.9 using the Nanopore preset parameters and an estimated genome size of 5 Mb [62]. Resulting contigs were binned with Concoct v1.1.0 [63], MetaBAT v2 [64], and MaxBin v2.2.7 [54]. Bins were refined using the *bin_refinement* module of MetaWrap v1.2.1 [65]. Illumina reads were mapped to each bin using Bowtie v2.3.4.1 [49], and Nanopore reads were mapped to each bin using Minimap v2.17 [66]. Mapped Illumina and Nanopore reads for each bin were used for hybrid assembly using Unicycler v0.4.8 [67]. Resulting MAGs were refined with refineM [61] as described above and the incongruence of contig and 16S rRNA gene taxonomy was checked manually. Refined Illumina-based and hybrid MAGs were characterized with CheckM and QUAST. MAGs were annotated using Prokka v1.13 [68] and Prodigal v2.6.3 [69,70]. Taxonomy was assigned with GTDB-tk v1.0.2 using GTDB-Tk reference data version r89 [71]. Additionally, the hybrid MAGs were analyzed using the type (strain) genome server of DSMZ—German Collection of Microorganisms and Cell Cultures GmbH (https://tygs.dsmz.de/user_requests/new, accessed on 11 July 2018). dRep [72] was used to identify representative MAGs after the refinement step. Lastly, refined and de-replicated MAGs were obtained and analyzed.

Finally, the HUMAnN 2.0 pipeline [73] was used to test for the presence of selected pathways [74] in the Prodigal-annotated hybrid MAGs. Coding sequences were mapped against the UniRef50 EC filtered database using DIAMOND. Default parameter settings were used except for lowering the translated query coverage threshold from 90.0 to 50.0 to account for coding sequences being longer than sequencing reads, which are the typical input for HUMAnN 2.0. Identified gene families were mapped to EggNOG COGs using the humann2_regroup_table script.

Metagenome data of MiSeq Illumina sequencing, Oxford Nanopore sequencing, and representative MAGs are available at https://www.ebi.ac.uk/ena/browser/view/PRJEB42791.

## 3. Results

### 3.1. Impact of Feeding Regime and OLR on Microbial Community Composition

After quality-filtering, between 46% and 67% of all reads could be aligned to the database of expected species. Assembly and binning of unaligned reads led to eleven bins with a contamination below 5% and a completeness higher than 95%. After reassembly, the average total assembly length (mean over the total number of contigs over all samples) was around 26 Mb, the longest contig was around 347 kb, and the highest N50 value was 7.5 kb over all samples (see Supplementary File S2 for details).

**Figure 1:**
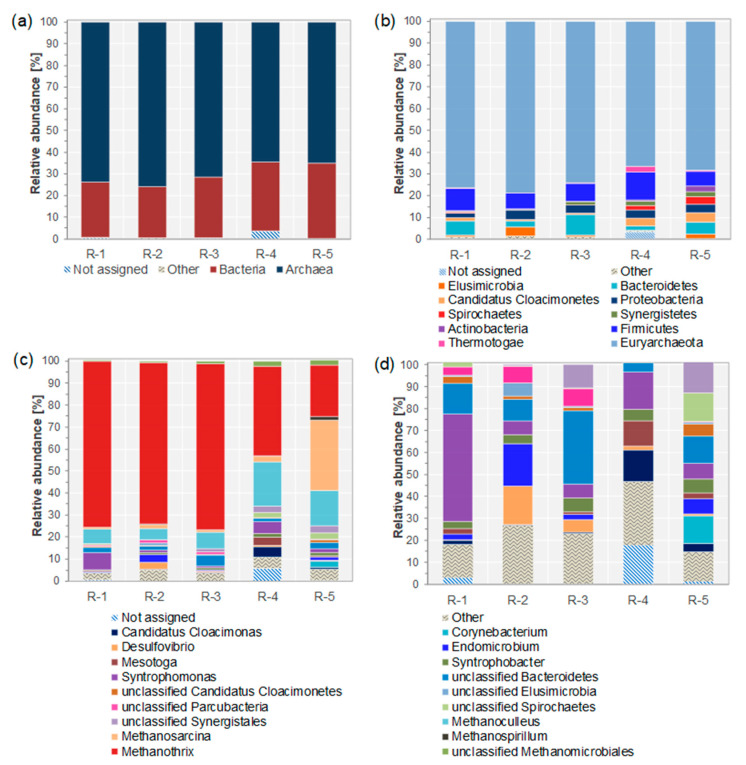
Microbial community composition at domain (**a**), phylum (**b**), and genus level (**c**), and composition of the bacterial communities only at genus level (**d**). Relative abundances were calculated using read counts per taxon. In all panels, not assigned refers to reads without hits on the respective taxonomic level as well as unclassified reads. Other refers to reads assigned to other domains (**a**), phyla, respectively, genera, with relative abundances below 1% (**b**,**c**), and bacterial genera with relative abundances below 5% (**d**). Panels (**c**,**d**) share the same legend.

The microbial communities in all reactors were dominated by archaea (around 70% relative abundance, see Figure 1a). R-1, R-2, and R-3 revealed similar archaeal community compositions with comparable relative abundances (see Figure 1c). *Methanothrix* had a relative abundance of around 75% and *Methanoculleus* of at least 6%, whereas *Methanosarcina* was less abundant (around 1%). R-4 showed differences in terms of a lower relative abundance of *Methanothrix* (41%) and a higher relative abundance of *Methanoculleus* (20%) compared to R-1, R-2, and R-3. *Methanosarina* increased in abundance up to around 3% in R-4. More conspicuous was the higher relative abundance of *Methanosarcina* in R-5, with around 32%.

R-4 and R-5 showed higher proportions of bacteria (around 33%) than R-1, R-2, and R-3 (around 26%, Figure 1b). Nine bacterial phyla constituted the bacterial community, with *Firmicutes* dominating in all reactors (between around 7% and 13%). *Bacteroidetes* were more abundant in R-1, R-2, and R-3 compared to R-4 and R-5, with around 6.5% compared to 3.8% relative abundance. Besides *Spirochaetes*, *Cloacimonetes* were more abundant in R-4 and R-5 than in R-1, R-2, and R-3. Comparing the bacterial communities on the genus level (Figure 1c,d), *Desulfovibrio* only contributed significantly to the bacterial community composition in discontinuously fed reactors with low OLR (R-2 and R-3). *Mesotoga* reached up to around 12% but only under continuous feeding at high OLR. At low OLR under continuous feeding (R-1), *Syntrophomonas* was the dominant genus, while at discontinuous feeding or higher OLR (R-2 to R-5), its relative abundance was lower. Under low OLR, either one or two bacterial genera contributed more than 30% of the bacterial composition (*Syntrophomonas* under continuous feeding, and either unclassified Bacteroidetes or *Desulfovibrio* with *Endomicrobium* under discontinuous feeding), while at high OLR, the distribution was more even. For more information regarding other taxonomic levels, see Supplementary File S2, B.2.1.

### 3.2. Combining Taxonomic and Functional Annotation

**Figure 2:**
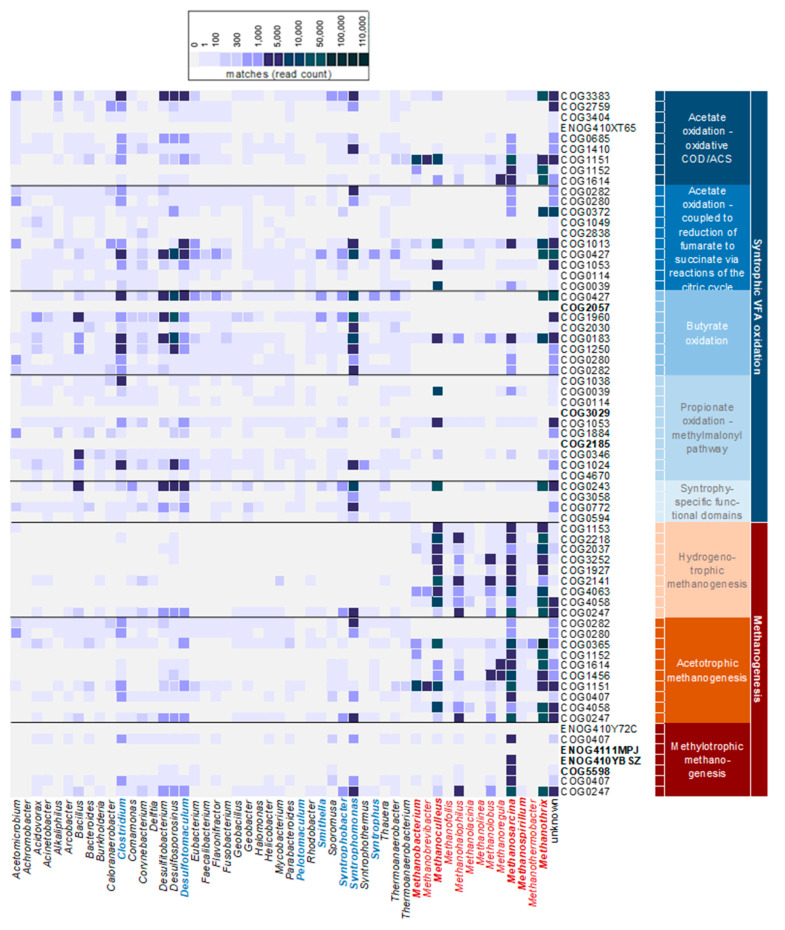
Taxonomic and functional annotation of metagenome reads. Rows contain pathway-specific genes for propionate, butyrate, and acetate degradation as well as methanogenesis pathways. The color gradient represents read abundance (read counts per genus and potential function as absolute values). Genera with most matches for the selected pathways are shown. IDs in bold indicate alternatives to searched gene IDs, or were not available (see Supplementary File S1). Genera indicated in blue were associated with SCFA oxidation pathways and genera indicated in red with methanogenesis pathways. The “unknown” section represents all reads that were not assigned to any known organism in the NCBI database.

Compared to PCR-dependent analysis methods, metagenomes enable the analysis of community composition without PCR bias, and additionally reveal the functional potential. Even though the bacterial community composition was different in the five reactors, the functional potential was very similar (see Supplementary File S2, B.2.2). Therefore, annotation results were merged over all reactors to assess the functional potential of individual taxa by combining functional and taxonomic annotation of metagenomic reads. For this analysis, 7000 genes and 3500 taxa were considered (see Supplementary File S2, B.4). As SCFAs were supplied as the sole carbon source, the further analysis focused on syntrophic SCFA oxidation and methanogenesis pathways (see Figure 2 and Supplementary File S2, B.5).

Nine genes selected for the oxidative carbon monoxide dehydrogenase/acetyl-CoA synthase (CODH/ACS) pathway (Supplementary File S1) of acetate oxidation were detected and seven of nine genes were unique for the CODH/ACS pathway. Only *Acetomicrobium* covered the whole CODH/ACS pathway. A high coverage of this pathway was shown for *Clostridium* and *Desulfotomaculum*. The acetate oxidation pathway coupled to the reduction of fumarate (Supplementary File S1) was almost completely covered by *Burkholderia*, *Corynebacterium*, and *Geobacter*.

Except for the missing COG2057 gene (acetate CoA-transferase beta subunit), *Syntrophomonas* and *Clostridium* covered all selected genes for butyrate oxidation (Supplementary File S1). However, many other genera covered almost the whole butyrate oxidation pathway as well, for example, *Bacillus*, *Desulfotomaculum*, *Corynebacterium*, and *Faecalibacterium*, among others.

None of the bacterial taxa covered the whole propionate oxidation pathway (Supplementary File S1). The bacterial genera *Bacillus*, *Clostridium*, *Desulfotomaculum*, and *Pelotomaculum* covered a great part of the propionate oxidation pathway to a lesser extent (around eight of ten selected genes). Accordingly, expected SPOB like *Smithella* and *Syntrophobacter* were rare as reflected by their low relative abundance in the whole community.

Syntrophy-specific functional domains of butyrate or propionate oxidation, such as the extra-cytoplasmic formate dehydrogenase (FDH), FDH accessory protein, membrane-integrated proteins, and ribonuclease (Supplementary File S1) were completely encoded in *Syntrophobacter*, *Syntrophomonas*, and *Syntrophus* as well as in *Bacillus* and *Desulfotomaculum*.

In our analysis, *Clostridium* showed a high functional potential and gene coverage in all pathways of acetogenesis (Figure 2). This is in line with *Clostridium* species having been reported as versatile contributors in the processes of AD [75,76].

The hydrogenotrophic methanogenesis pathway was completely covered by *Methanosarcina*, *Methanoculleus*, *Methanothrix*, *Methanolobus*, *Methanohalophilus*, and *Methanospirillum* (Figure 2). The acetotrophic methanogenesis pathway was almost completely covered by *Methanosarcina* and partially by *Methanothrix*, *Methanohalophilus*, and *Methanoculleus*. The acetate-CoA ligase (COG0365) was encoded in all archaeal genera. Additionally, *Methanosarcina* also covered the methylotrophic methanogenesis pathway. Therefore, the functional potential of *Methanosarcina* for all pathways of methanogenesis was detected.

### 3.3. Metagenome-Assembled Genomes

#### 3.3.1. MAGs Reconstructed from Illumina Reads Only

MAGs were reconstructed and analyzed to elucidate the functional role of the detected species in more detail. In total, 94 MAGs were obtained. After refinement, 27 high-quality and 29 medium-quality MAGs were generated (see Table 2), from which 45 bacterial MAGs and 11 archaeal MAGs were allocated (see Supplementary File S2, B.6.1 for details). The archaeal MAGs were mainly assigned to the genera *Methanothrix*, *Methanoculleus*, and *Methanospirillum*. The identified bacterial MAGs were taxonomically more diverse and assigned to several already described MAGs (listed in the GTDB). All results of MAG reconstruction from Illumina MiSeq reads are listed in detail in Supplementary File S2, B.6.1. The mean N50 value was 32.24 kb, the total assembly length around 2.29 Mb, and the largest contig was 93.70 kb.

#### 3.3.2. MAGs Based on the Hybrid Assembly Approach

The Illumina MAG analysis was extended by including Oxford Nanopore reads for the samples from the continuously fed reactors (R-1 and R-4, see Supplementary File S2, B.6.2). In total, 27 MAGs based on the hybrid approach were obtained for both reactors, including 13 high-quality and four medium-quality MAGs. Thirteen of these MAGs could be matched to known species (Table 3). Three high-quality archaeal MAGs were obtained with a completeness between 97.22% and 99.35%, and 0.65% contamination (Table 3). Two of them represented *Methanothrix soehngenii* and the third one *Methanoculleus*. Additionally, ten high-quality bacterial MAGs were reconstructed, see Supplementary File S2, B.6.2. A *Cloacimonas acidaminovorans* MAG had the highest quality and was circular. Six of the 13 dereplicated MAGs showed a close relationship to *Syntrophobacteraceae* or *Syntrophomonas*. Four MAGs represented circular genomes of *Methanothrix soehngenii*, *Syntrophomonas wolfei*, *Cloacimonas acidaminovorans*, and *Mesotoga infera* B. The mean N50 value was 1.7 Mb, the total assembly length around 3 Mb, and the largest contig was 1.9 Mb.

#### 3.3.3. Pathways of Interest Encoded by MAGs

**Figure 3:**
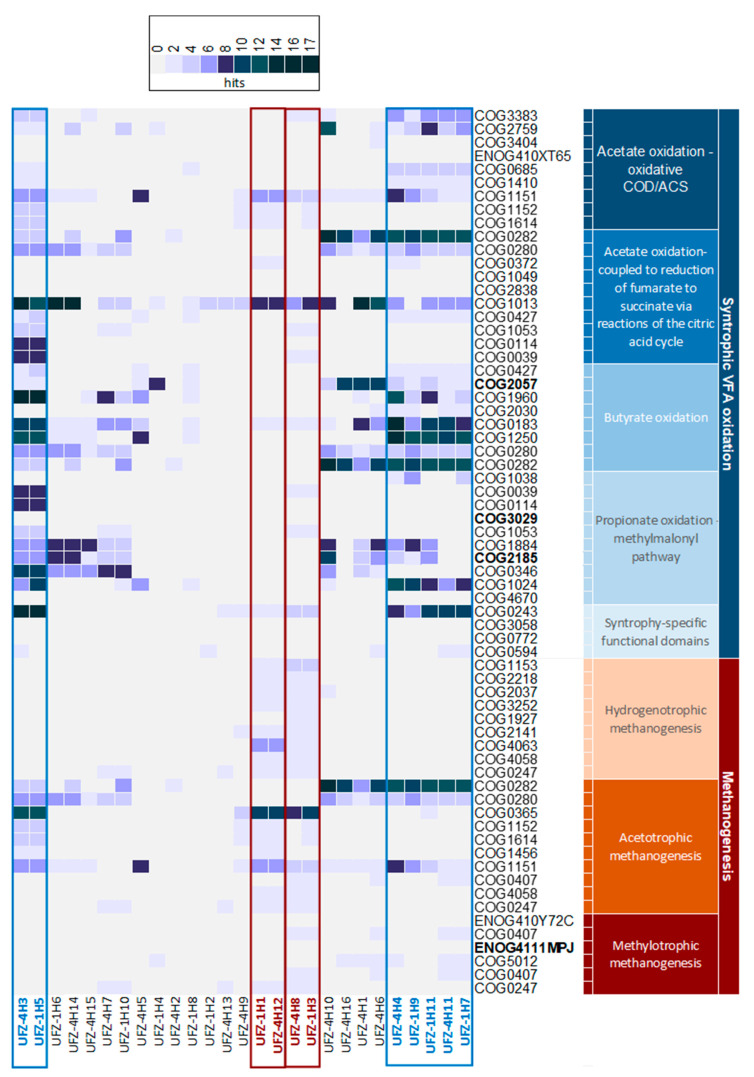
Pathway analysis of hybrid assembly MAGs. “Hits” refers to the number of genes per MAG and pathway. Rows contain selected genes of pathways of interest. For detailed information, the COG/EggNOG-IDs are listed in Supplementary File S1. IDs in bold indicate alternatives to searched gene IDs, or were not available (see Supplementary File S1). MAGs indicated in blue show similar patterns in syntrophic SCFA oxidation. MAGs indicated in red show similar patterns in methanogenesis.

To elucidate the functional role of organisms represented by the reconstructed MAGs within the community, individual MAGs were tested for the presence of genes encoding syntrophic SCFA degradation and methanogenesis pathways (Figure 3). Four clusters of similar enzyme repertoires were identified. Both members of the first cluster (UFZ-4H3 and UFZ-1H5) were assigned to the family *Syntrophobacteraceae*. They contained genes of all SCFA oxidation pathways including the almost complete pathways for syntrophic butyrate and propionate oxidation. Two MAGs (UFZ-1H1 and UFZ-4H12), both identified as *Methanothrix soehngenii*, formed the second cluster and covered a great part of the hydrogenotrophic methanogenesis pathway (eight out of nine genes). Both MAGs contained six of the seven unique genes for hydrogenotrophic methanogenesis (encoding formylmethanofuran dehydrogenase, formylmethanofuran-H_4_MPT formyltransferase, methenyl-H_4_MPT cyclohydrolase, H_2_-forming methylene-H_4_MPT dehydrogenase, and F_420_-dependent methylene-H_4_MPT reductase). Hydrogenotrophic methanogenesis was fully covered in the third cluster (UFZ-1H3 and UFZ-4H8) with both MAGs being assigned to *Methanoculleus*. The last cluster contained five MAGs (UFZ-4H4, UFZ-1H9, UFZ-1H11, UFZ-4H11, and UFZ-1H7) that were all assigned to the family *Syntrophomonadaceae*. All enzymes belonging to butyrate degradation were found to be encoded in these MAGs (Figure 3).

## 4. Discussion

By using metagenome approaches, we analyzed composition and functional potential of microbial communities in anaerobic digesters under two different feeding regimes at high and low OLRs, with a focus on the conversion of SCFAs to methane. We focused the analysis on selected pathways of particular interest (SCFA oxidation and methanogenesis) and detected associated genes in reads and MAGs. MAGs were reconstructed and annotated to shed light on uncharacterized taxa. The goal was to characterize the full community and identify the putative functional role of not yet described species.

Previous analysis of the methanogenic reactor communities by *mcrA*-gene based terminal restriction fragment length polymorphism (T-RFLP) analysis had identified *Methanothrix* and *Methanomicrobiaceae* as the main constituents, and an increased share of *Methanosarcina* under discontinuous feeding [1]. These findings were confirmed with metagenome-based analysis in which *Methanothrix* and *Methanoculleus* were the main archaeal genera, and the share of *Methanosarcina* was increased under discontinuous feeding (R-2, R-3, R-5, Figure 1c), a shift that was more pronounced under high OLRs (R-5, [1]), where *Methansarcina* partially replaced *Methanothrix*. It is known that *Methanothrix* is more sensitive to changing environmental conditions and stress conditions (feeding regime or OLR) than *Methanosarcina* and *Methanoculleus* [5,77,78,79,80]. Hence, stress tolerance of AD systems can be promoted if *Methanosarcina* can be enriched in the reactor at the expense of the more sensitive species, increasing the functional resilience of the methane production process [1]. Discontinuous feeding was shown to increase the relative abundance of *Methanosarcina*, and Bonk et al. [1] hypothesized that the dynamic environment established by discontinuous feeding provides temporal niches that enable the observed shift in the archaeal community.

The bacterial communities were unique for all reactors, even for reactors R-2 and R-3, which were biological replicates, and no clear trends could be observed regarding OLR and feeding regime (Figure 1d). This has been reported before, including similar values for diversity and evenness of the bacterial communities based on 16S rRNA gene amplicon sequencing data [1].

Using metagenomic data to assess community composition circumvents any PCR biases and, by targeting bacteria and archaea simultaneously, enables the assessment of the ratio between both domains. The observed dominance of archaea was expected as the direct supply of SCFAs as substrate led to the establishment of only the last two steps of AD (i.e., syntrophic SCFA oxidation and methanogenesis), excluding the hydrolysis and acidogenesis steps driven by bacteria. Comparable ratios of bacteria to archaea were also found in other anaerobic digesters supplied with SCFAs [81]. Propionate and butyrate are degraded by bacteria in syntrophy with hydrogenotrophic methanogens, while acetate can either be directly utilized by acetoclastic methanogens or by acetate-oxidizing bacteria, again requiring a hydrogenotrophic methanogen as a syntrophic partner.

Despite the observed variability in bacterial community composition, the overall reactor productivity was not affected, indicating the presence of functional redundancy in the bacterial communities. In low OLR reactors, the bacterial community was dominated by one or two bacterial genera, while high OLR reactors showed a more even distribution of the different genera, indicating that competition played a bigger role under nutrient-limiting conditions. An adaptation of the bacterial community due to an increase in OLR was also observed by Maus et al. [82].

Although being dominated by archaeal genera, the reactors in this study harboured diverse bacterial genera, many with relative abundances below 1%. Given that SCFAs were provided as the sole carbon sources, one could expect that most of these genera are involved in syntrophic SCFA oxidation. *Syntrophobacter* and *Pelotomaculum* were identified as known syntrophic oxidizers for propionate. *Synthrophomonas* was the only butyrate-oxidizing genus detected. Genera such as *Cloacimonetes* [83,84,85], *Cryptanaerobacter* [86], and *Desulfovibrio* [87] have been associated with the oxidation of propionic or butyric acid. *Candidatus* Cloacamonas acidaminovorans, for example, contains all the genes for propionic acid oxidation via methylmalonyl-CoA, but has not yet been isolated [83].

Besides these known and putative SCFA-oxidizers, our MAGs analysis uncovered many bacteria that were apparently not involved in SCFA oxidation. Similar observations have been made in AD reactors being fed with a single SCFA (acetate or propionate) [88]. Yeast extract was present in the medium in the latter study, potentially supporting the growth of other bacteria than SCFA oxidizers. However, this supplement was absent in our study, and hence cannot explain the observed bacterial diversity. Bacteria not directly utilizing the primary carbon source could either be involved in biomass turnover as scavengers or thrive on proteins, amino acids, polysaccharides, and lipids produced by primary consumers and associated syntrophic partners. Autotrophic methanogens can actively excrete amino acids into the medium [75,76], and amino acid exchange has been postulated in a synthetic coculture [89]. Furthermore, metabolite cross-feeding, especially between phylogenetically distant species, might be an important factor driving microbial community dynamics [90], and explain the observed diversity of bacterial taxa, including proteolytic fermenters and amino acid oxidizers not involved in SCFA oxidation. The primary consumers together with their syntrophic partners may hence be the source of excreted nutrients, which sustain a low abundance shadow microbial community of taxa not involved in the primary process.

Overall, the microbial community composition at the genus level was more similar for reactors only differing in feeding regime, whereas those with different OLR harboured more distinct communities, indicating the importance of substrate quantity for shaping community composition. For example, the ratio of bacteria to archaea was mostly determined by the OLR but not by the feeding regime.

The functional potential was similar between reactors despite different community composition. Hence, functional redundancy in SCFA degradation by known or unknown bacterial species could play an important role. By applying a higher OLR (reactors R-4 and R-5), the relative abundance of bacteria increased compared to the low OLR reactors R-1 to R-3. However, the genus *Syntrophomonas*, comprising typical butyrate-oxidizing bacteria [20,91], showed the highest abundance in the low OLR reactor R-1. *Syntrophomonas* was also described as the major syntrophic butyrate-oxidizing bacterium in a butyrate-fed system [92]. Its lower abundance in the other reactors indicates that other species took over its function there. Other species only appeared under high OLR conditions or increased their abundance. For instance, *Corynebacterium* was not found in R-2 and R-3 but had an abundance in the bacterial community of around 13% in R-5, and *Mesotoga* was present with a relative abundance of less than 1% in R-2 and R-3 but reached around 3% in R-5. Genera like unclassified *Bacteroidetes*, *Candidatus* Cloacimonas, *Mesotoga, Desulfovibrio*, or *Endomicrobium* were detected with higher abundance, but none of these taxa covered the complete butyrate degradation pathway. Pathway analysis showed that *Desulfosporosinus*, *Clostridium*, or *Bacillus* showed the same pathway coverage of selected genes as *Syntrophomonas*. However, the relative abundance of these genera was below 1% of the whole community. Thus, potential functional redundancy between yet undescribed taxa and known taxa might be present in our reactors. *Candidatus* Cloacimonas was detected mainly in the R-4 community with around 5%. Being frequently detected in AD processes [83,93], the reconstruction of a high quality MAG classified as *Candidatus* Cloacimonas and with 100% completeness (UFZ-4H1) provides the opportunity to identify its role in the process. However, regarding the main pathways analyzed in this study, there was no clear indication for its participation in syntrophic SCFA oxidation. For example, only three genes out of ten for propionate oxidation via the methonylmalonyl-CoA pathway were found (Figure 3), although the complete pathway was reported for the genome of *Candidatus* C. aminoacidivorans [83].

Regarding typical propionate-degrading bacteria like *Desulfotomaculum*, *Pelotomaculum*, *Smithella*, and *Syntrophobacter* [20], only *Syntrophobacter* was detected with more than 5% in the bacterial community and only around 1.5% in the whole community of R-4 and R-5. The pathway analysis of selected genes of propionate oxidation showed a low coverage of these genes by *Syntrophobacter*. In contrast, *Clostridium* and *Desulfotomaculum* displayed a higher share of the typical propionate oxidation genes. A closer examination of *Desulfotomaculum* suggested that this genus is metabolically versatile, because it encodes many selected genes for acetate, butyrate and propionate oxidation. A single species of *Desulfotomaculum* has been described as syntrophic propionate oxidizer yet [94].

The results of Illumina reads as well as the analysis of MAGs showed that *Methanothrix* (UFZ-4H12) encoded all selected genes of the hydrogenotrophic methanogenesis pathway, besides the acetoclastic methanogenesis pathway. This finding was described already by Smith & Ingram-Smith [95]. As hydrogenotrophic methanogenesis activity of *Methanothrix* has not been described, it has been speculated that this genus can produce methane based on direct interspecies electron transfer [96]. However, as acetic acid was available in our reactors, *Methanothrix* most likely utilized the acetoclastic methanogenesis pathway.

Read length is a limitation of the Illumina platform but the high quality is an advantage. Oxford Nanopore reads are long but the quality with an error rate up to 15% is much higher compared to the Illumina read quality [32,33,34]. By applying a hybrid assembly approach, the disadvantages of both techniques can be compensated. Even though Nanopore reads get more accurate due to improvements in technology, Illumina, for polishing of especially low-coverage MAGs, is advantageous [97].

We were expecting that the completeness of the genomes would increase compared to Illumina short read-based MAGs. Furthermore, as expected and as described in the literature e.g., [38,97] the contiguity of the MAGs was considerably increased by incorporating long reads.

By the reconstruction of MAGs, several potentially new species could be identified. Focusing on high quality MAGs, a *Syntrophobacteraceae* species potentially involved in syntrophic SCFA oxidation (UFZ-4H3), three *Syntrophomonadaceae* species potentially involved in butyrate oxidation (UFZ-4H4, UFZ-1H11, and UFZ-1H7), and one *Methanoculleus* species potentially involved in hydrogenotrophic methanogenesis (UFZ-1H3) were identified (Figure 3). The metabolic pathway analysis focused on the two key processes in the reactor: syntrophic SCFA oxidation as the utilization step of the provided carbon sources and methanogenesis as the syntrophically coupled process, providing the required electron sink. However, some of the high-quality MAGs could not be clearly assigned to these key processes (UFZ-4H14, UFZ-1H10, UFZ-4H10, UFZ-4H1, and UFZ-4H6; Figure 3). This indicates that the core community catalyzing the key processes is able to support a wider food web, explaining the observed high diversity. The discovered MAGs provide a first glimpse into this food web, potentially comprising metabolite cross-feeding and scavengers involved in biomass turnover.

The high relative abundance of the archaeal populations limited the read numbers for bacterial populations, limiting the opportunity to reconstruct complete bacterial genomes of rare species. A higher number of bacterial reads would allow to reconstruct more complete genomes of potential new functional groups or species. Nevertheless, 13 medium- and high-quality MAGs could be obtained. The GTDBbk database classification leads to the assumption that some bacterial MAGs belong to the families *Syntrophobacteraceae* (UFZ-4H3, UFZ-4H4) and *Cloacimonadaceae* (UFZ-4H1) or the order *Bacteroidales (UFZ-1H10)*.

Concluding, the hybrid assembly revealed to be the optimal approach for genome reconstruction, because the advantage of quality (Illumina approach) and read length (Nanopore) and disadvantages of short reads (Illumina) and lower quality (Nanopore) complement each other. Illumina-based sequencing approaches usually lead to highly fragmented MAGs. This means that complex genomes can only rarely be reconstructed. The functional understanding of microbiomes from AD systems could be expanded and more information could be retrieved from metagenomic samples. After all, an approach that uses a dereplicated set of recovered MAGs from hybrid assemblies is likely to improve our ability to reconstruct genomes and can even enable the strain-specific reconstruction of genomes from metagenomic data.

## Figures and Tables

**Table 1:** Experimental setup and process parameters according to [1]. For discontinuously fed reactors, measurements were taken just prior to feeding; mean values and standard deviation are reported for the stable operating phase (29 days for R-1, R-2, R-3, and 44 days for R-4, R-5) with daily measurements of pH and methane production and weekly measurements of total SCFA and microbial biomass (see Figure S1). COD: chemical oxygen demand; VS: volatile solids.

	R-1	R-2	R-3	R-4	R-5
**OLR (gCOD/L/d)**	1.55			4.65	
**Feeding regime**	Continuous feeding over 24 h	75% daily feed at once, 25% continuously		Continuous feeding over 24 h	100% daily feed at once
**Replicates**	--	Biological	Biological	--	--
**pH**	7.5 ± 0.1	7.5 ± 0.1	7.5 ± 0.1	7.2 ± 0.1	7.5 ± 0.2
**Total SCFA concentration (mg/L)**	86.9 ± 28.7	338.8 ± 413.5	56.5 ± 45.2	195.6 ± 371.3	304.0 ± 455.6
**Methane production rate (L/d)**	3.8 ± 0.5	4.1 ± 0.4	4.4 ± 0.3	11.8 ± 2.9	10.5 ± 1.9
**Microbial biomass (gVS/L)**	0.37 ± 0.01	0.34 ± 0.07	0.34 ± 0.05	0.96 ± 0.15	0.79 ± 0.07

**Table 2:** Summary of Illumina-based MAGs after refinement.

Quality	ID	Completeness [%]	Contamination [%]	Length [Mb]	Taxonomic Classification According to GTDB
HQ	UFZ-5_008	100	1.69	2	*g__58-81 (f_Thermovirgaceae)*
HQ	UFZ-1_001	99.35	0.65	2.8	*s__Methanothrix soehngenii*
HQ	UFZ-2_001	99.35	0.65	2.8	*s__Methanothrix soehngenii*
HQ	UFZ-3_001	99.35	0.65	2.8	*s__Methanothrix soehngenii*
HQ	UFZ-4_002	99.35	0.65	2.7	*s__Methanothrix soehngenii*
HQ	UFZ-5_003	99.35	1.31	2.7	*s__Methanothrix soehngenii*
HQ	UFZ-4_007	99.15	3.54	2	*g__58-81 (f_Thermovirgaceae)*
MQ	UFZ-5_013	97.9	6.37	4.1	*f__Syntrophobacteraceae*
HQ	UFZ-1_009	97.8	0	2.2	*g__UBA3900*
HQ	UFZ-5_005	97.8	0	2.1	*g__UBA3900 (f_Cloacimonadaceae)*
HQ	UFZ-1_013	97.75	1.56	1.7	*f__Endomicrobiaceae*
HQ	UFZ-2_003	97.19	0.37	1.7	*f__Endomicrobiaceae*
HQ	UFZ-3_003	96.68	4.39	3.7	*s__UBA5314 sp002410325 (f_Syntrophomonadaceae)*
HQ	UFZ-5_009	96.07	0.44	1.6	*f__Endomicrobiaceae*
MQ	UFZ-4_004	95.82	5.82	3.4	*s__UBA5314 sp002410325 (f_Syntrophomonadaceae)*
HQ	UFZ-5_004	95.42	0.65	3.8	*s__Methanosarcina mazei*
MQ	UFZ-2_004	95.15	6.13	3.8	*s__UBA5314 sp002410325 (f_Syntrophomonadaceae)*
HQ	UFZ-3_007	94.76	1.85	1.7	*g__58-81 (f_Thermovirgaceae)*
HQ	UFZ-2_002	94.56	1.72	2.7	*g__Methanoculleus*
HQ	UFZ-4_001	94.44	2.75	2.7	*g__Methanoculleus*
HQ	UFZ-4_008	94.25	1.79	2.4	*s__UBA2256 sp001603165 (o_Treponematales)*
HQ	UFZ-5_007	94.25	3.58	2.5	*s__UBA2256 sp001603165 (o_Treponematales)*
HQ	UFZ-5_010	93.47	3.14	2.5	*f__4484-276 (o_Bacteroidales)*
HQ	UFZ-1_010	93.37	1.09	2.6	*f__Syntrophomonadaceae*
HQ	UFZ-1_014	93.1	2.98	2.4	*s__UBA2256 sp001603165 (o_Treponematales)*
HQ	UFZ-1_018	93.04	4.25	4	*f__Syntrophobacteraceae*
HQ	UFZ-4_011	92.42	2.32	4	*f__Syntrophobacteraceae*
HQ	UFZ-5_002	92.19	3.16	2.4	*g__Methanoculleus*
HQ	UFZ-3_004	92.17	4.4	2.5	*f__4484-276 (o_Bacteroidales)*
HQ	UFZ-1_004	91.73	0.21	2.5	*s__Syntrophomonas wolfei*
MQ	UFZ-3_002	89.58	5.28	2.7	*g__Methanoculleus*
MQ	UFZ-1_019	87.85	2.59	2.7	*s__Mesotoga sp002305955*
MQ	UFZ-1_003	86.8	2.69	2.1	*g__Methanoculleus*
MQ	UFZ-1_020	86.69	3.12	2.7	*s__UBA5314 sp002410325 (f_Syntrophomonadaceae)*
MQ	UFZ-3_005	86.67	1.11	2.8	*f__BBW3 (o_Bacteroidales)*
MQ	UFZ-1_006	86.27	0.54	2.6	*f__4484-276 (o_Bacteroidales)*
MQ	UFZ-3_009	86.13	9.59	3.7	*f__Syntrophobacteraceae*
MQ	UFZ-5_018	83.06	2.72	1.9	*g__Syntrophomonas_B*
MQ	UFZ-4_003	80.88	0.47	2.3	*s__Mesotoga infera_B*
MQ	UFZ-2_008	72.42	1.61	2.2	*f__BBW3 (o_Bacteroidales)*
MQ	UFZ-5_012	71.28	1.19	2	*g__Corynebacterium*
MQ	UFZ-5_014	68.95	2.01	1.3	*g__Methanospirillum*
MQ	UFZ-5_017	68.02	3.11	1.8	*g__SR-FBR-E99 (o_Bacteroidales)*
MQ	UFZ-4_010	67.63	0.86	1.7	*f__4484-276 (o_Bacteroidales)*
MQ	UFZ-4_006	67.58	4.4	1.3	*s__Cloacimonas acidaminovorans*
MQ	UFZ-5_015	67.1	3.22	1.2	*f__DTU023 (o_Saccharofermentanales)*
MQ	UFZ-2_006	63.94	1.34	1.7	*f__4484-276 (o_Bacteroidales)*
MQ	UFZ-2_005	62.8	1.12	0.4	*s__UBA2558 sp002340425 (o_Paceibacterales)*
MQ	UFZ-4_012	62.67	1.21	1.4	*p__Firmicutes_A*
MQ	UFZ-3_008	60.22	2.88	0.9	*f__Endomicrobiaceae*
MQ	UFZ-5_019	56.14	5.65	1.4	*f__Salinivirgaceae*
MQ	UFZ-2_007	55.22	1.66	1.3	*g__Methanoculleus*
MQ	UFZ-1_029	55.05	2	0.9	*g__58-81 (f_Thermovirgaceae)*
MQ	UFZ-5_006	53.45	0	1.6	*-*
MQ	UFZ-2_010	50.76	2.01	1	*g__UBA3900 (f_Cloacimonadaceae)*
MQ	UFZ-2_009	50.08	0.22	2.1	*f__Syntrophobacteraceae*

HQ: high quality MAGs with >90% completeness and <5% contamination, MQ: medium quality MAGs with >50% completeness and <10% contamination; p: phylum; o: order; f: family; g: genus; and s: species.

**Table 3:** Summary of results of hybrid assembly-based MAGs after refinement.

Quality	ID	Completeness [%]	Contamination [%]	Length [Mb]	GTDB Classification	TYGS Classification *
HQ	UFZ-4H1	100	1.1	2.2	*s__Cloacimonas acidaminovorans*	Potential new species
HQ	UFZ-4H6	99.84	0.47	3.0	*s__Mesotoga infera_B*	*Mesotoga infera*
HQ	UFZ-4H12	99.35	0.65	2.9	*s__Methanothrix soehngenii*	*Methanothrix soehngenii*
HQ	UFZ-1H11	98.98	1.86	3.2	*g__DTU019 (f_Syntrophomonadaceae)*	Potential new species
HQ	UFZ-4H3	98.87	2.1	4.4	*f__Syntrophobacteraceae*	Potential new species
HQ	UFZ-4H4	98.09	4.67	4.4	*s__UBA5314 sp002410325 (f_Syntrophobacteraceae)*	*Desulfovibrio paquesii*
HQ	UFZ-1H7	97.86	1.23	3.0	*s__Syntrophomonas wolfei*	*Syntrophomonas wolfei*
HQ	UFZ-1H3	97.22	0.65	2.9	*g__Methanoculleus*	Potential new species
HQ	UFZ-4H14	96.55	0	2.7	*s__UBA2256 sp001603165 (o_Treponematales*)	Potential new species
HQ	UFZ-4H10	95.84	1.69	2.2	*g__58-81 (f_Thermovirgaceae)*	Potential new species
MQ	UFZ-4H11	91.99	7.01	3.4	*s__Syntrophomonas wolfei*	Potential new species
HQ	UFZ-1H10	90.59	0.54	2.7	*f__4484-476 (o_Bacteroidales*)	Potential new species
MQ	UFZ-1H9	78.19	0.58	1.8	*g__Syntrophomonas_B*	Potential new species

* https://tygs.dsmz.de/user_requests/new, accessed on 11 July 2018. HQ: high quality MAGs with >90% completeness and <5% contamination, MQ: medium quality MAGs with >50% completeness and <10% contamination; o: order; f: family; g: genus; and s: species.

## Data Availability

The sequence data have been deposited in the ENA database under BioProject PRJEB42791 (https://www.ebi.ac.uk/ena/data/view/PRJEB42791).

## Supplementary Materials

The following supporting information can be downloaded at https://www.mdpi.com/article/10.3390/microorganisms11020420/s1: Supplementary File S1 (PDF file) containing details on experimental design, reactor performance and enzyme-pathway mappings and Supplementary File S2 (Excel file) containing additional sequencing analysis data.

## References

[1] Bonk F., Popp D., Weinrich S., Sträuber H., Kleinsteuber S., Harms H., Centler F. (2018). Intermittent Fasting for Microbes: How Discontinuous Feeding Increases Functional Stability in Anaerobic Digestion. Biotechnol. Biofuels.

[2] Vanwonterghem I., Jensen P.D., Dennis P.G., Hugenholtz P., Rabaey K., Tyson G.W. (2014). Deterministic Processes Guide Long-Term Synchronised Population Dynamics in Replicate Anaerobic Digesters. ISME J..

[3] Falk M.W., Song K.-G., Matiasek M.G., Wuertz S. (2009). Microbial Community Dynamics in Replicate Membrane Bioreactors—Natural Reproducible Fluctuations. Water Res..

[4] Mulat D.G., Jacobi H.F., Feilberg A., Adamsen A.P.S., Richnow H.-H., Nikolausz M. (2016). Changing Feeding Regimes to Demonstrate Flexible Biogas Production: Effects on Process Performance, Microbial Community Structure, and Methanogenesis Pathways. Appl. Env. Microb..

[5] Vrieze J.D., Hennebel T., Boon N., Verstraete W. (2012). *Methanosarcina*: The Rediscovered Methanogen for Heavy Duty Biomethanation. Bioresour. Technol..

[6] Ziels R.M., Beck D.A.C., Stensel H.D. (2017). Long-Chain Fatty Acid Feeding Frequency in Anaerobic Codigestion Impacts Syntrophic Community Structure and Biokinetics. Water Res..

[7] Mauky E., Jacobi H.F., Liebetrau J., Nelles M. (2015). Flexible Biogas Production for Demand-Driven Energy Supply—Feeding Strategies and Types of Substrates. Bioresour. Technol..

[8] Vrieze J.D., Verstraete W., Boon N. (2013). Repeated Pulse Feeding Induces Functional Stability in Anaerobic Digestion. Microb. Biotechnol..

[9] Willeghems G., Buysse J. (2016). Changing Old Habits: The Case of Feeding Patterns in Anaerobic Digesters. Renew. Energ..

[10] Li Y., Ma J., Yuan H., Li X. (2022). Effects of Feeding Regimes on Process Performance and Microbial Community Structure in Anaerobic Semi-Continuously Stirred Tank Reactors Treating Corn Stover. Waste Biomass Valorization.

[11] Gerardi M.H. (2003). The Microbiology of Anaerobic Digesters. Wastewater Microbiology Series.

[12] Hao L., Michaelsen T.Y., Singleton C.M., Dottorini G., Kirkegaard R.H., Albertsen M., Nielsen P.H., Dueholm M.S. (2020). Novel Syntrophic Bacteria in Full-Scale Anaerobic Digesters Revealed by Genome-Centric Metatranscriptomics. ISME J..

[13] Kirkegaard R.H., McIlroy S.J., Kristensen J.M., Nierychlo M., Karst S.M., Dueholm M.S., Albertsen M., Nielsen P.H. (2017). The Impact of Immigration on Microbial Community Composition in Full-Scale Anaerobic Digesters. Sci. Rep..

[14] Ahring B.K., Sandberg M., Angelidaki I. (1995). Volatile Fatty Acids as Indicators of Process Imbalance in Anaerobic Digestors. Appl. Microbiol. Biot..

[15] Boe K., Batstone D.J., Steyer J.-P., Angelidaki I. (2010). State Indicators for Monitoring the Anaerobic Digestion Process. Water Res..

[16] Arumugam K., Bessarab I., Haryono M.A.S., Liu X., Zuniga–Montanez R.E., Roy S., Qiu G., Drautz–Moses D.I., Law Y.Y., Wuertz S. (2021). Recovery of Complete Genomes and Non-Chromosomal Replicons from Activated Sludge Enrichment Microbial Communities with Long Read Metagenome Sequencing. Npj Biofilms Microbiomes.

[17] McInerney M.J., Struchtemeyer C.G., Sieber J., Mouttaki H., Stams A.J.M., Schink B., Rohlin L., Gunsalus R.P. (2008). Physiology, Ecology, Phylogeny, and Genomics of Microorganisms Capable of Syntrophic Metabolism. Ann. NY Acad. Sci..

[18] Sousa D.Z., Smidt H., Alves M.M., Stams A.J.M. (2009). Ecophysiology of Syntrophic Communities That Degrade Saturated and Unsaturated Long-Chain Fatty Acids: Ecophysiology of Syntrophic LCFA Degradation. FEMS Microbiol. Ecol..

[19] Manzoor S., Schnürer A., Bongcam-Rudloff E., Müller B. (2018). Genome-Guided Analysis of Clostridium ultunense and Comparative Genomics Reveal Different Strategies for Acetate Oxidation and Energy Conservation in Syntrophic Acetate-Oxidising Bacteria. Genes.

[20] Müller B., Sun L., Schnürer A. (2013). First Insights into the Syntrophic Acetate-oxidizing Bacteria—A Genetic Study. MicrobiologyOpen.

[21] Westerholm M., Moestedt J., Schnürer A. (2016). Biogas Production through Syntrophic Acetate Oxidation and Deliberate Operating Strategies for Improved Digester Performance. Appl. Energ..

[22] Hattori S. (2008). Syntrophic Acetate-Oxidizing Microbes in Methanogenic Environments. Microbes Environ..

[23] Plugge C.M., Henstra A.M., Worm P., Swarts D.C., Paulitsch-Fuchs A.H., Scholten J.C.M., Lykidis A., Lapidus A.L., Goltsman E., Kim E. (2012). Complete Genome Sequence of *Syntrophobacter fumaroxidans* Strain (MPOB(T)). Stand. Genom. Sci..

[24] Felchner-Zwirello M., Winter J., Gallert C. (2013). Interspecies Distances between Propionic Acid Degraders and Methanogens in Syntrophic Consortia for Optimal Hydrogen Transfer. Appl. Microbiol. Biot..

[25] Plugge C.M., Dijkema C., Stams A.J.M. (1993). Acetyl-CoA Cleavage Pathway in a Syntrophic Propionate Oxidizing Bacterium Growing on Fumarate in the Absence of Methanogens. FEMS Microbiol. Lett..

[26] Bok F.A.M., de Stams A.J.M., Dijkema C., Boone D.R. (2001). Pathway of Propionate Oxidation by a Syntrophic Culture of *Smithella propionica* and *Methanospirillum hungatei*. Appl. Environ. Microb..

[27] Knoop F. (1904). Der Abbau aromatischer Fettsäuren im Tierkörper. Beitr. Chem. Physiol. Pathol..

[28] Enzmann F., Mayer F., Rother M., Holtmann D. (2018). Methanogens: Biochemical Background and Biotechnological Applications. AMB Express.

[29] Kurth J.M., Camp H.J.M.O., den Welte C.U. (2020). Several Ways One Goal—Methanogenesis from Unconventional Substrates. Appl. Microbiol. Biot..

[30] Crable B.R., Plugge C.M., McInerney M.J., Stams A.J.M. (2011). Formate Formation and Formate Conversion in Biological Fuels Production. Enzym. Res..

[31] Sikora A., Detman A., Mielecki D., Chojnacka A., Błaszczyk M., Banu J.R. (2018). Searching for Metabolic Pathways of Anaerobic Digestion: A Useful List of the Key Enzymes. Anaerobic Digestion.

[32] Quick J., Quinlan A.R., Loman N.J. (2014). A Reference Bacterial Genome Dataset Generated on the MinION^TM^ Portable Single-Molecule Nanopore Sequencer. Gigascience.

[33] Quince C., Walker A.W., Simpson J.T., Loman N.J., Segata N. (2017). Shotgun Metagenomics, from Sampling to Analysis. Nat. Biotechnol..

[34] Salmela L., Walve R., Rivals E., Ukkonen E. (2017). Accurate Self-Correction of Errors in Long Reads Using de Bruijn Graphs. Bioinformatics.

[35] Stoler N., Nekrutenko A. (2021). Sequencing Error Profiles of Illumina Sequencing Instruments. NAR Genom. Bioinform..

[36] Damme R.V., Hölzer M., Viehweger A., Müller B., Bongcam-Rudloff E., Brandt C. (2021). Metagenomics Workflow for Hybrid Assembly, Differential Coverage Binning, Metatranscriptomics and Pathway Analysis (MUFFIN). PLoS Comput. Biol..

[37] Liu L., Wang Y., Yang Y., Wang D., Cheng S.H., Zheng C., Zhang T. (2021). Charting the Complexity of the Activated Sludge Microbiome through a Hybrid Sequencing Strategy. Microbiome.

[38] Tedersoo L., Albertsen M., Anslan S., Callahan B. (2021). Perspectives and Benefits of High-Throughput Long-Read Sequencing in Microbial Ecology. Appl. Environ. Microb..

[39] Arumugam K., Bessarab I., Haryono M.A.S., Liu X., Zuniga-Montanez R.E., Roy S., Qiu G., Drautz-Moses D.I., Law Y.Y., Wuertz S. (2020). Analysis Procedures for Assessing Recovery of High Quality, Complete, Closed Genomes from Nanopore Long Read Metagenome Sequencing. BioRxiv.

[40] Lui L.M., Nielsen T.N., Arkin A.P. (2021). A Method for Achieving Complete Microbial Genomes and Improving Bins from Metagenomics Data. PLoS Comput. Biol..

[41] Moss E.L., Maghini D.G., Bhatt A.S. (2020). Complete, Closed Bacterial Genomes from Microbiomes Using Nanopore Sequencing. Nat. Biotechnol..

[42] Cerdán J.M.A., Tejido-Nuñez Y., Aymerich E., de GoñiGoñi J.G.-M., Garcia-Aguirre J. (2021). A Comprehensive Comparison of Methane and Bio-Based Volatile Fatty Acids Production from Urban and Agro-Industrial Sources. Waste Biomass Valorization.

[43] Bolger A.M., Lohse M., Usadel B. (2014). Trimmomatic: A Flexible Trimmer for Illumina Sequence Data. Bioinformatics.

[44] Andrews S. FastQC: A Quality Control Tool for High Throughput Sequence Data [Online]. Http://www.Bioinformatics.Babraham.Ac.Uk/Projects/Fastqc/.

[45] Ewels P., Magnusson M., Lundin S., Kaller M. (2016). MultiQC: Summarize Analysis Results for Multiple Tools and Samples in a Single Report. Bioinformatics.

[46] Becker D., Popp D., Harms H., Centler F. (2020). A Modular Metagenomics Pipeline Allowing for the Inclusion of Prior Knowledge Using the Example of Anaerobic Digestion. Microorganisms.

[47] Bengtsson-Palme J., Hartmann M., Eriksson K.M., Pal C., Thorell K., Larsson D.G.J., Nilsson R.H. (2015). Metaxa2: Improved Identification and Taxonomic Classification of Small and Large Subunit rRNA in Metagenomic Data. Mol. Ecol. Resour..

[48] Bengtsson J., Eriksson K.M., Hartmann M., Wang Z., Shenoy B.D., Grelet G.-A., Abarenkov K., Petri A., Rosenblad M.A., Nilsson R.H. (2011). Metaxa: A Software Tool for Automated Detection and Discrimination among Ribosomal Small Subunit (12S/16S/18S) Sequences of Archaea, Bacteria, Eukaryotes, Mitochondria, and Chloroplasts in Metagenomes and Environmental Sequencing Datasets. Antonie Van Leeuwenhoek.

[49] Langmead B., Salzberg S.L. (2012). Fast Gapped-Read Alignment with Bowtie 2. Nat. Methods.

[50] Buchfink B., Xie C., Huson D.H. (2015). Fast and Sensitive Protein Alignment Using DIAMOND. Nat. Methods.

[51] Huson D.H., Beier S., Flade I., Górska A., El-Hadidi M., Mitra S., Ruscheweyh H.-J., Tappu R. (2016). MEGAN Community Edition—Interactive Exploration and Analysis of Large-Scale Microbiome Sequencing Data. PLoS Comput. Biol..

[52] Peng Y., Leung H.C.M., Yiu S.M., Chin F.Y.L. (2012). IDBA-UD: A de Novo Assembler for Single-Cell and Metagenomic Sequencing Data with Highly Uneven Depth. Bioinformatics.

[53] Peng Y., Leung H.C.M., Yiu S.M., Chin F.Y.L. (2010). Research in Computational Molecular Biology. The Lecture Notes in Computer Science (Including Subseries Lecture Notes in Artificial Intelligence and Lecture Notes in Bioinformatics, Proceedings of the 14th Annual International Conference, RECOMB 2010, Lisbon, Portugal, 25–28 April 2010.

[54] Wu Y.-W., Simmons B.A., Singer S.W. (2016). MaxBin 2.0: An Automated Binning Algorithm to Recover Genomes from Multiple Metagenomic Datasets. Bioinformatics.

[55] Wu Y.-W., Tang Y.-H., Tringe S.G., Simmons B.A., Singer S.W. (2014). MaxBin: An Automated Binning Method to Recover Individual Genomes from Metagenomes Using an Expectation-Maximization Algorithm. Microbiome.

[56] Gurevich A., Saveliev V., Vyahhi N., Tesler G. (2013). QUAST: Quality Assessment Tool for Genome Assemblies. Bioinformatics.

[57] Parks D.H., Imelfort M., Skennerton C.T., Hugenholtz P., Tyson G.W. (2015). CheckM: Assessing the Quality of Microbial Genomes Recovered from Isolates, Single Cells, and Metagenomes. Genome Res..

[58] Huerta-Cepas J., Szklarczyk D., Forslund K., Cook H., Heller D., Walter M.C., Rattei T., Mende D.R., Sunagawa S., Kuhn M. (2016). EggNOG 4.5: A Hierarchical Orthology Framework with Improved Functional Annotations for Eukaryotic, Prokaryotic and Viral Sequences. Nucleic Acids Res..

[59] Nurk S., Meleshko D., Korobeynikov A., Pevzner P.A. (2017). MetaSPAdes: A New Versatile Metagenomic Assembler. Genome Res..

[60] Bowers R.M., Kyrpides N.C., Stepanauskas R., Harmon-Smith M., Doud D., Reddy T.B.K., Schulz F., Jarett J., Rivers A.R., Eloe-Fadrosh E.A. (2017). Minimum Information about a Single Amplified Genome (MISAG) and a Metagenome-Assembled Genome (MIMAG) of Bacteria and Archaea. Nat. Biotechnol..

[61] Parks D.H., Rinke C., Chuvochina M., Chaumeil P.-A., Woodcroft B.J., Evans P.N., Hugenholtz P., Tyson G.W. (2017). Recovery of Nearly 8,000 Metagenome-Assembled Genomes Substantially Expands the Tree of Life. Nat. Microbiol..

[62] Koren S., Walenz B.P., Berlin K., Miller J.R., Bergman N.H., Phillippy A.M. (2017). Canu: Scalable and Accurate Long-Read Assembly via Adaptive k-Mer Weighting and Repeat Separation. Genome Res..

[63] Alneberg J., Bjarnason B.S., de Bruijn I., Schirmer M., Quick J., Ijaz U.Z., Lahti L., Loman N.J., Andersson A.F., Quince C. (2014). Binning Metagenomic Contigs by Coverage and Composition. Nat. Methods.

[64] Kang D.D., Li F., Kirton E., Thomas A., Egan R., An H., Wang Z. (2019). MetaBAT 2: An Adaptive Binning Algorithm for Robust and Efficient Genome Reconstruction from Metagenome Assemblies. PeerJ.

[65] Uritskiy G.V., DiRuggiero J., Taylor J. (2018). MetaWRAP—A Flexible Pipeline for Genome-Resolved Metagenomic Data Analysis. Microbiome.

[66] Li H. (2018). Minimap2: Pairwise Alignment for Nucleotide Sequences. Bioinformatics.

[67] Wick R.R., Judd L.M., Gorrie C.L., Holt K.E. (2017). Unicycler: Resolving Bacterial Genome Assemblies from Short and Long Sequencing Reads. PLoS Comput. Biol..

[68] Seemann T. (2014). Prokka: Rapid Prokaryotic Genome Annotation. Bioinformatics.

[69] Hyatt D., Chen G.-L., LoCascio P.F., Land M.L., Larimer F.W., Hauser L.J. (2010). Prodigal: Prokaryotic Gene Recognition and Translation Initiation Site Identification. BMC Bioinform..

[70] Hyatt D., LoCascio P.F., Hauser L.J., Uberbacher E.C. (2012). Gene and Translation Initiation Site Prediction in Metagenomic Sequences. Bioinformatics.

[71] Chaumeil P.-A., Mussig A.J., Hugenholtz P., Parks D.H. (2019). GTDB-Tk: A Toolkit to Classify Genomes with the Genome Taxonomy Database. Bioinformatics.

[72] Olm M.R., Brown C.T., Brooks B., Banfield J.F. (2017). DRep: A Tool for Fast and Accurate Genomic Comparisons That Enables Improved Genome Recovery from Metagenomes through de-Replication. ISME J..

[73] Franzosa E.A., McIver L.J., Rahnavard G., Thompson L.R., Schirmer M., Weingart G., Lipson K.S., Knight R., Caporaso J.G., Segata N. (2018). Species-Level Functional Profiling of Metagenomes and Metatranscriptomes. Nat. Methods.

[74] Caspi R., Billington R., Keseler I.M., Kothari A., Krummenacker M., Midford P.E., Ong W.K., Paley S., Subhraveti P., Karp P.D. (2019). The MetaCyc Database of Metabolic Pathways and Enzymes—A 2019 Update. Nucleic Acids Res..

[75] Shen J., Yu Z., Zhu W. (2018). Insights into the Populations of Proteolytic and Amino Acid-Fermenting Bacteria from Microbiota Analysis Using In Vitro Enrichment Cultures. Curr. Microbiol..

[76] Örlygsson J., Krooneman J., Collins M.D., Pascual C., Gottschal J.C. (1996). *Clostridium acetireducens* sp. nov., a Novel Amino Acid-Oxidizing, Acetate-Reducing Anaerobic Bacterium. Int. J. Syst. Evol. Microbiol..

[77] Calli B., Mertoglu B., Inanc B., Yenigun O. (2005). Community Changes During Start-up in Methanogenic Bioreactors Exposed to Increasing Levels of Ammonia. Environ. Technol..

[78] Sprott G.D., Patel G.B. (1986). Ammonia Toxicity in Pure Cultures of Methanogenic Bacteria. Syst. Appl. Microbiol..

[79] Steinhaus B., Garcia M.L., Shen A.Q., Angenent L.T. (2007). A Portable Anaerobic Microbioreactor Reveals Optimum Growth Conditions for the Methanogen *Methanosaeta concilii*. Appl. Environ. Microb..

[80] Klang J., Theuerl S., Szewzyk U., Huth M., Tölle R., Klocke M. (2015). Dynamic Variation of the Microbial Community Structure during the Long-time Mono-fermentation of Maize and Sugar Beet Silage. Microb. Biotechnol..

[81] Campanaro S., Treu L., Rodriguez-R L.M., Kovalovszki A., Ziels R.M., Maus I., Zhu X., Kougias P.G., Basile A., Luo G. (2020). New Insights from the Biogas Microbiome by Comprehensive Genome-Resolved Metagenomics of Nearly 1600 Species Originating from Multiple Anaerobic Digesters. Biotechnol. Biofuels.

[82] Maus I., Klocke M., Derenkó J., Stolze Y., Beckstette M., Jost C., Wibberg D., Blom J., Henke C., Willenbücher K. (2020). Impact of Process Temperature and Organic Loading Rate on Cellulolytic/Hydrolytic Biofilm Microbiomes during Biomethanation of Ryegrass Silage Revealed by Genome-Centered Metagenomics and Metatranscriptomics. Environ. Microbiome.

[83] Pelletier E., Kreimeyer A., Bocs S., Rouy Z., Gyapay G., Chouari R., Rivière D., Ganesan A., Daegelen P., Sghir A. (2008). “Candidatus Cloacamonas acidaminovorans”: Genome Sequence Reconstruction Provides a First Glimpse of a New Bacterial Division. J. Bacteriol..

[84] Juste-Poinapen N.M.S., Turner M.S., Rabaey K., Virdis B., Batstone D.J. (2015). Evaluating the Potential Impact of Proton Carriers on Syntrophic Propionate Oxidation. Sci. Rep..

[85] Ahlert S., Zimmermann R., Ebling J., König H. (2016). Analysis of Propionate-degrading Consortia from Agricultural Biogas Plants. Microbiologyopen.

[86] De Bok F.A.M., Harmsen H.J.M., Plugge C.M., de Vries M.C., Akkermans A.D.L., de Vos W.M., Stams A.J.M. (2005). The First True Obligately Syntrophic Propionate-Oxidizing Bacterium, *Pelotomaculum schinkii* sp. nov., Co-Cultured with Methanospirillum hungatei, and Emended Description of the Genus Pelotomaculum. Int. J. Syst. Evol. Microbiol..

[87] Suzuki D., Ueki A., Shizuku T., Ohtaki Y., Ueki K. (2010). *Desulfovibrio butyratiphilus* sp. nov., a Gram-Negative, Butyrate-Oxidizing, Sulfate-Reducing Bacterium Isolated from an Anaerobic Municipal Sewage Sludge Digester. Int. J. Syst. Evol. Microbiol..

[88] Singh A., Schnürer A., Westerholm M. (2021). Enrichment and Description of Novel Bacteria Performing Syntrophic Propionate Oxidation at High Ammonia Level. Environ. Microbiol.

[89] Moreira J.P.C., Diender M., Arantes A.L., Boeren S., Stams A.J.M., Alves M.M., Alves J.I., Sousa D.Z. (2021). Propionate Production from Carbon Monoxide by Synthetic Cocultures of *Acetobacterium wieringae* and *Propionigenic bacteria*. Appl. Environ. Microb..

[90] Giri S., Oña L., Waschina S., Shitut S., Yousif G., Kaleta C., Kost C. (2021). Metabolic Dissimilarity Determines the Establishment of Cross-Feeding Interactions in Bacteria. Curr. Biol..

[91] Wallrabenstein C., Schink B. (1994). Evidence of Reversed Electron Transport in Syntrophic Butyrate or Benzoate Oxidation by *Syntrophomonas wolfei* and *Syntrophus buswellii*. Arch. Microbiol..

[92] Meng X., Cao Q., Sun Y., Huang S., Liu X., Li D. (2022). 16S rRNA Genes- and Metagenome-Based Confirmation of Syntrophic Butyrate-Oxidizing Methanogenesis Enriched in High Butyrate Loading. Bioresour. Technol..

[93] Solli L., Håvelsrud O.E., Horn S.J., Rike A.G. (2014). A Metagenomic Study of the Microbial Communities in Four Parallel Biogas Reactors. Biotechnol. Biofuels.

[94] Plugge C.M., Balk M., Stams A.J.M. (2002). *Desulfotomaculum thermobenzoicum* subsp. *Thermosyntrophicum* subsp. nov., a Thermophilic, Syntrophic, Propionate-Oxidizing, Spore-Forming Bacterium. Int. J. Syst. Evol. Microbiol..

[95] Smith K.S., Ingram-Smith C. (2007). Methanosaeta, the Forgotten Methanogen?. Trends Microbiol..

[96] Rotaru A.-E., Shrestha P.M., Liu F., Shrestha M., Shrestha D., Embree M., Zengler K., Wardman C., Nevin K.P., Lovley D.R. (2013). A New Model for Electron Flow during Anaerobic Digestion: Direct Interspecies Electron Transfer to Methanosaeta for the Reduction of Carbon Dioxide to Methane. Energ. Environ. Sci..

[97] Sereika M., Kirkegaard R.H., Karst S.M., Michaelsen T.Y., Sørensen E.A., Wollenberg R.D., Albertsen M. (2022). Oxford Nanopore R10.4 Long-Read Sequencing Enables the Generation of near-Finished Bacterial Genomes from Pure Cultures and Metagenomes without Short-Read or Reference Polishing. Nat. Methods.

