# Supplementary material for: Metagenomic Analysis of Anaerobic Microbial Communities Degrading Short-Chain Fatty Acids as Sole Carbon Sources
Source: Microorganisms. 2023 Feb 7;11(2):420. doi: 10.3390/microorganisms11020420 (PMC9959488; doi:10.3390/microorganisms11020420)
Supplement: Supplementary file 1 [file microorganisms-11-00420-s001.zip › Supplementary File 1.pdf]

Metagenomic analysis of anaerobic microbial communities degrading short-chain fatty acids as sole carbon sources

Daniela Becker [1,2], Denny Popp [1,3], Fabian Bonk [1,4], Sabine Kleinsteuber [1], Hauke Harms [1] and Florian Centler [1,5]*

[1] UFZ – Helmholtz Centre for Environmental Research, Department of Environmental Microbiology, Permoserstr. 15, 04318 Leipzig, Germany
[2] Current address: IAV GmbH, Kauffahrtei 23-25, 09120 Chemnitz, Germany
[3] Current address: Institute of Human Genetics, University of Leipzig Medical Center, Philipp-Rosenthal-Str. 55, 04103 Leipzig, Germany
[4] Current address: VERBIO Vereinigte Bioenergie AG, Thura Mark 18, 06780 Zörbig, Germany
[5] Current address: School of Life Sciences, University of Siegen, 57076 Siegen, Germany
* Correspondence: florian.centler@uni-siegen.de

Supplementary File 1

1. Experimental setup

In this study, a metagenomic approach was applied to explore the microbial communities and functional profiles of AD reactors established in a previous experimental study [1]. Briefly, five reactors were operated under different feeding regimes (continuous and discontinuous) and organic loading rates (OLRs) with a supply of SCFAs (acetate, propionate and butyrate) as the sole carbon sources (Table 1 in main text). Hence, we focused on acetogenesis and methanogenesis, which are often bottlenecks of the AD process. The continuously fed reactors and the discontinuously fed reactors were operated at a hydraulic retention time of 8 days and at two different OLRs (1.55 gCOD/L/d and 4.65 gCOD/L/d). The latter value corresponds to 3.4 gVS/L/d and hence represents a typical agricultural biogas plant with a reported average OLR of 3.2 gVS/L/d (https://biogas.fnr.de/daten-und-fakten/faustzahlen, accessed on February 17, 2022), while the former represents a less intensely operated biogas plant. Discontinuous feeding was implemented by applying a singular daily feed pulse (Table 1). For details, see Bonk et al. [1]. Reactors R-2 and R-3 were replicate reactors (biological replicates) with identical process conditions and feeding regimes. Reactors R-1 to R-3 correspond to Experiment 3 described in Bonk et al. [1], and R-4 and R-5 to Experiment 2.

2. Process Performance

All reactors reached a stable production phase after the initial startup phase (Figure S1). All low OLR reactors (R-1, R-2, R-3) had a methane production rate between 3.9 and 4.5 L/d in this phase, while both high OLR reactors (R-4, R-5) achieved 10 and 12.6 L/d (Table 1). The average SCFA concentration in all reactors was below 0.36 gCOD/L before feeding. Regardless of the feeding regime (continuous or discontinuous with one pulse feed per day), pH value, methane production rate and microbial biomass concentration were similar (Figure S1), reaching a substrate conversion efficiency of more than 99% [1]. However, an increased OLR led to higher and more fluctuating methane production, more biomass in the reactors, and a slightly decreased pH value. Table 1 in the main text summarizes the process parameters as determined in the last measurement before feeding.

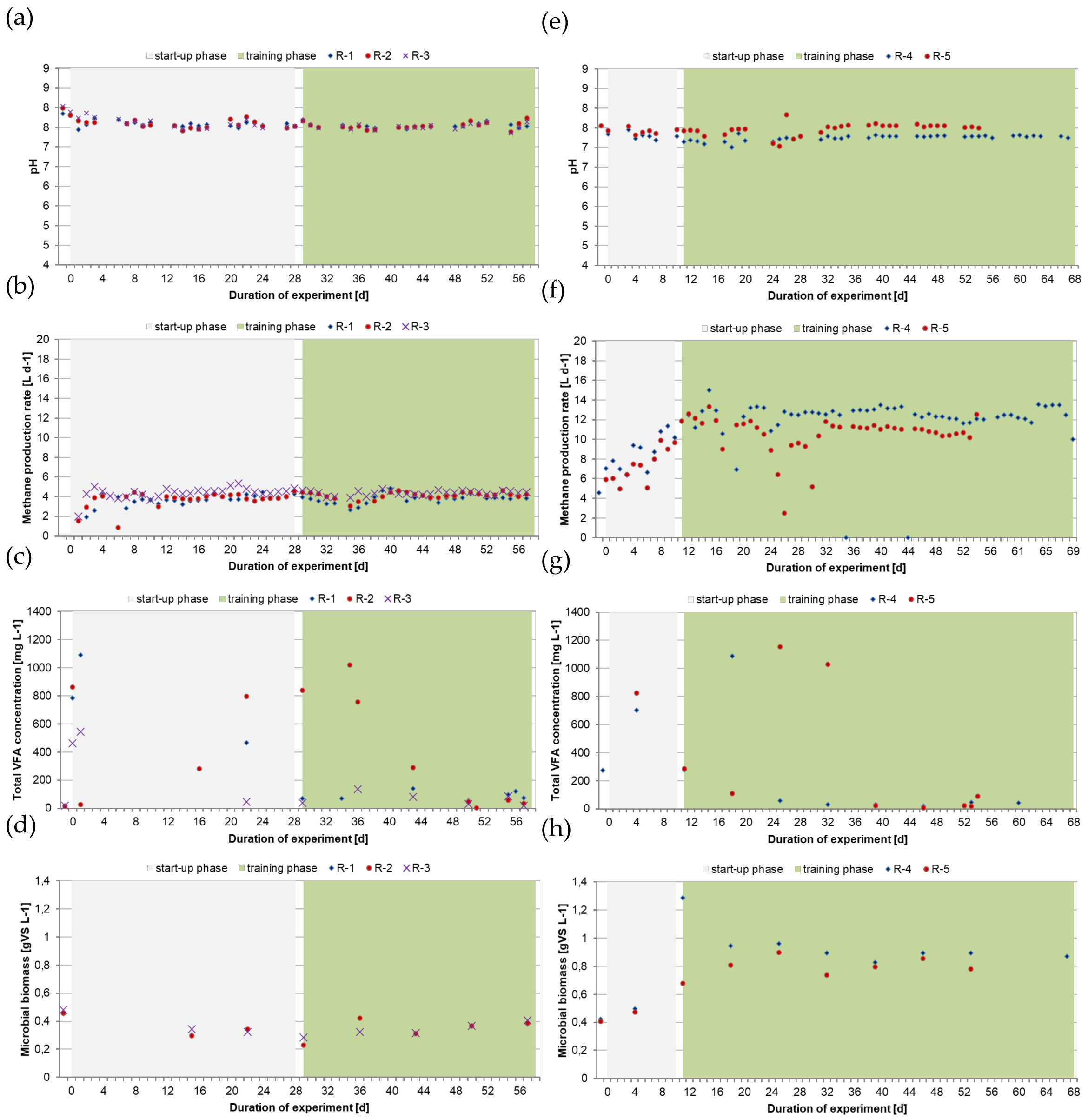

Figure S1. Process performance during bioreactor experiments performed earlier [1]. While feeding of the continuously fed reactors R-1 and R-4 was identical in start-up and training phase, for discontinuously fed reactors R-2, R-3, and R-5, feeding was ramped up in the start-up phase and kept constant throughout the training phase to enable the microbial reactor community to adapt to the pulsed feeding regime. Reactors R-1, R-2, and R-3 received an organic loading rate of 1.55 gCOD/L/d (**a**-**d**), while R-4 and R-5 received 4.65 gCOD/L/d (**e**-**h**). Temporal evolution of pH (**a**,**e**), methane production rate (**b**,**f**), total volatile fatty acid (VFA) concentration (**c**,**g**), and microbial biomass concentration (**d**,**h**). Sampling for this study occurred on day 58 for reactors R-1, R-2, and R-3, and on day 54 for reactors R-4 and R-5.

3. Representative genes for pathways

Representative genes for selected pathways were taken from [2].

Table S1. SCFA (short-chain fatty acid) oxidation pathways, bold entries indicate non-unique enzymes.

Pathway	Enzyme	EC number	COG	EggNOG	UniProt

**Acetate oxidation** - oxidative carbon monoxide dehydrogenase/acetyl-CoA synthase	NADP-dependent formate dehydrogenase	EC 1.17.1.10	COG3383	ENOG4108HIZ	P77908
	Formyltetrahydrofolate synthetase	EC 6.3.4.3	COG2759	ENOG4105CKU	P21164
	Methenyltetrahydrofolate cyclohydrolase	EC 3.5.4.9	COG3404	ENOG410908R	Q49135
	NADP-dependent methylenetetrahydrofolate dehydrogenase	EC 1.5.1.5	ENOG410XT65	ENOG4108N1H	P55818
	Ferredoxin-dependent methylenetetrahydrofolate reductase	EC 1.5.7.1			
	5,10-Methylenetetrahydrofolate reductase	EC 1.5.1.20	COG0685	ENOG4105SYT	P0AEZ1
	5-Methyltetrahydrofolate:corrinoid/iron-sulfur protein Co-methyltransferase	EC 2.1.1.258	COG1410	ENOG4107VP5	Q46389
	Carbon monoxide dehydrogenase	**EC 1.2.7.4**	**COG1151**	**ENOG4105CJG**	P31896
	CO-methylating acetyl-CoA synthase	**EC 2.3.1.169**	COG1152	**ENOG4105E8X**	P27988
			COG1614		
**Acetate oxidation** - reduction of fumarate	Acetate kinase	**EC 2.7.2.1**	**COG0282**	**ENOG4105C6H**	P71104
	Phosphotransacetylase	**EC 2.3.1.8**	**COG0280**	**ENOG4108IJ2**	A5N801
	Citrate synthase	EC 2.3.3.1	COG0372	ENOG4105BZN	P0ABH7
	Aconitase	EC 4.2.1.3	COG1049	ENOG4107QIJ	P56418
	Isocitrate dehydrogenase (NADP+-dependent)	EC1.1.1.42	COG2838	ENOG4105E9K	P16100
	2-Oxoglutarate:ferredoxin oxidoreductase	EC 1.2.7.3	COG1013	ENOG4105DQU	O53181
	Succinyl-CoA:acetate-CoA transferase	EC 2.8.3.18	**COG0427**	**ENOG4105CYC**	P38946
	Succinate dehydrogenase	**EC 1.3.5.1**	**COG1053**	**ENOG4105C00**	O53370
	Fumarate hydratase	**EC 4.2.1.2**	**COG0114**	**KOG1317**	P07954
	Malate dehydrogenase	**EC 1.1.1.37**	**COG0039**	**ENOG4105C80**	P61889
**Butyrate oxidation**	CoA transferase	EC 2.8.3.9	**COG0427**	**ENOG4105CYC**	P33752
	Acetate-CoA transferase beta-subunit		COG2057	ENOG4107QNJ	P76459
	Butyryl-CoA dehydrogenase	EC 1.3.8.1	COG1960	ENOG4105C1G	A5N5C8
	Crotonase-3-OH-butyryl-CoA dehydratase (3-hydroxybutyryl-CoA dehydratase)	EC 4.2.1.55	COG2030	ENOG4108F01	C5AZ74
	Acetyl-CoA acetyltransferase	EC 2.3.1.9	COG0183	ENOG4105CHU	P76461
	Hydroxybutyryl-CoA dehydrogenase	EC 1.1.1.157	COG1250	ENOG4105DYT	P9WNP7
	Phosphotransacetylase	**EC 2.3.1.8**	**COG0280**	**ENOG4108IJ2**	A5N801
	Acetate kinase	**EC 2.7.2.1**	**COG0282**	**ENOG4105C6H**	P71104
**Propionate oxidation**	Pyruvate carboxylase	EC 6.4.1.1	COG1038	KOG0369	O93918
	Malate dehydrogenase	**EC 1.1.1.37**	**COG0039**	**ENOG4105C80**	P61889
	Fumarate hydratase	**EC 4.2.1.2**	**COG0114**	**KOG1317**	P07954
	Fumarate reductase	EC 1.3.5.4	COG3029	ENOG4108WJH	P0A8Q0
		EC 1.3.1.6			
	Succinate dehydrogenase	**EC 1.3.5.1**	**COG1053**	**ENOG4105C00**	O53370
	Succinyl-CoA synthetase	EC 6.2.1.4			
	Methylmalonyl-CoA mutase	EC 5.4.99.2	COG1884	ENOG4105D5P	P27253
			COG2185		

	Methylmalonyl-CoA epimerase	EC 5.1.99.1	COG0346	ENOG4108ZVT	Q8VQN0
	Methylmalonyl-CoA decarboxylase	EC 4.1.1.41 (new 7.2.4.3)	COG1024	ENOG4108M7F	P52045
	Propionate-CoA transferase	EC 2.8.3.1	COG4670	ENOG4108IIG	A0AKS6
**Syntrophy-specific functional domains of butyrate or propionate oxidation**	Extra-cytoplasmic formate dehydrogenase (FDH) alpha subunit, EC 1.17.1.9	EC 1.17.1.9	COG0243	ENOG4107QIW	P32176
	FdhE-like protein—tightly connected with FDH				
	FDH accessory protein—tightly connected with FDH		COG3058	ENOG4105F46	A0A066T1C1
	CapA—a membrane-bound complex, a protein involved in capsule or biofilm formation that may facilitate syntrophic growth (also present in acetate oxidizers)				P19579
	FtsW, RodA, SpoVE—membrane-integrated proteins involved in membrane integration, cell division, sporulation, and shape determination		**COG0772**	**ENOG4105CNI**	P0ABG4
			**COG0772**	**ENOG4105CNI**	P0ABG7
			**COG0772**	**ENOG4105CNI**	Q182Y5
	Ribonuclease P involved in tRNA maturation	EC 3.1.26.5	COG0594	ENOG4105NVJ	P0A7Y8

Table S2. Methanogenesis pathways, bold entries indicate non-unique enzymes.

**Hydrogenotrophic**	Formylmethanofuran dehydrogenase	EC 1.2.7.12	COG1153 and COG2218	arCOG00097	O27002
	Formylmethanofuran-H4MPT formyltransferase	EC 2.3.1.101	COG2037	arCOG02695	P55301
	Methenyl-H4MPT cyclohydrolase	EC 3.5.4.27	COG3252	ENOG41068T5	O85014
	F420-dependent methylene-H4MPT dehydrogenase	EC 1.5.98.1	COG1927	arCOG04382	P94951
	H2-forming methylene-H4MPT dehydrogenase	EC 1.12.98.2	COG4074 (alternative: COG1927)	arCOG03196	O27211
	F420-dependent methylene-H4MPT reductase	EC 1.5.98.2	COG2141	arCOG02410	O27784
	Methyl-H4MPT:coenzyme M methyl-transferase	EC 2.1.1.86	COG4063	arCOG03221	O59640
	Methyl-CoM reductase	**EC 2.8.4.1**	**COG4058**	**arCOG04857**	P07962
	Heterodisulfide reductase	**EC 1.8.98.1**	**COG0247**	**arCOG00333**	Q8PVW3
**Acetoclastic**	Acetate kinase (*Methanosarcina*)	**EC 2.7.2.1**	**COG0282**	**ENOG4105C6H**	P71104
	Phosphotransacetylase (*Methanosarcina*)	**EC 2.3.1.8**	**COG0280**	**ENOG4108IJ2**	A5N801
	Acetate-CoA ligase/acetate-CoA synthetase (*Methanosaeta*)	EC 6.2.1.1	COG0365	ENOG4108IQF	P27550
	CO-methylating acetyl-CoA synthase	**EC 2.3.1.169**	COG1152 and COG1614	**ENOG4105E8X**	P27988
	5-Methyl H4SPT:corrinoid/iron-sulfur protein Co-methyltransferase	EC 2.1.1.245	COG1456	arCOG01979	Q8PZ09
	Carbon monoxide dehydrogenase	**EC 1.2.7.4**	**COG1151**	**ENOG4105CJG**	P31896
	Methyl H4SPT:coenzyme M methyltransferase	EC 2.1.1.-	**COG0407**	**arCOG03323**	Q8TII4
	Methyl-CoM reductase	**EC 2.8.4.1**	**COG4058**	**arCOG04857**	P07962
	Heterodisulfide reductase	**EC 1.8.98.1**	**COG0247**	**arCOG00333**	Q8PVW3
**met**	Methanol:corrinoid protein Co-methyltransferase	EC 2.1.1.90	ENOG410Y72C	arCOG03330	Q46EH3

	[Methyl-Co(III) corrinoid protein]: coenzyme M methyltransferase	EC 2.1.1.246	**COG0407**	arCOG03324	Q48949
	Methylamine:corrinoid protein Co-methyltransferase	EC 2.1.1.248	ENOG4110FS3 (alternative: ENOG4111MPJ)	arCOG05143	O30642
	Dimethylamine:corrinoid protein Co-methyltransferase	**EC 2.1.1.249**	COG5012 (alternative: ENOG410YBSZ)	arCOG02030	O93657
	Trimethylamine:corrinoid protein Co-methyltransferase	**EC 2.1.1.249**	COG5012 (alternative: COG5598)	arCOG02028	O93659
	[Methyl-Co(III) methylamine-specific corrinoid protein]:coenzyme M methyltransferase	EC 2.1.1.247	**COG0407**	**arCOG03323**	O30640
	Methyl-CoM reductase	**EC 2.8.4.1**	**COG4058**	**arCOG04857**	P07962
	Heterodisulfide reductase	**EC 1.8.98.1**	**COG0247**	**arCOG00333**	Q8PVW3

References

1. Bonk, F.; Popp, D.; Weinrich, S.; Sträuber, H.; Kleinsteuber, S.; Harms, H.; Centler, F. Intermittent Fasting for Microbes: How Discontinuous Feeding Increases Functional Stability in Anaerobic Digestion. *Biotechnol Biofuels* 2018, *11*, 274, doi:10.1186/s13068-018-1279-5.

2. Sikora, A.; Detman, A.; Mielecki, D.; Chojnacka, A.; Błaszczyk, M. Searching for Metabolic Pathways of Anaerobic Digestion: A Useful List of the Key Enzymes. In *Anaerobic Digestion*; Banu, J.R., Ed.; 2018 ISBN 9781838818494.
